# HSPG-Deficient Zebrafish Uncovers Dental Aspect of Multiple Osteochondromas

**DOI:** 10.1371/journal.pone.0029734

**Published:** 2012-01-11

**Authors:** Malgorzata I. Wiweger, Zhe Zhao, Richard J. P. van Merkesteyn, Henry H. Roehl, Pancras C. W. Hogendoorn

**Affiliations:** 1 Department of Pathology, Leiden University Medical Centre, Leiden, The Netherlands; 2 Department of Oral and Maxillofacial Surgery, Leiden University Medical Centre, Leiden, The Netherlands; 3 Department of Biomedical Sciences, The University of Sheffield, Sheffield, United Kingdom; University of Western Ontario, Canada

## Abstract

Multiple Osteochondromas (MO; previously known as multiple hereditary exostosis) is an autosomal dominant genetic condition that is characterized by the formation of cartilaginous bone tumours (osteochondromas) at multiple sites in the skeleton, secondary bursa formation and impingement of nerves, tendons and vessels, bone curving, and short stature. MO is also known to be associated with arthritis, general pain, scarring and occasional malignant transformation of osteochondroma into secondary peripheral chondrosarcoma. MO patients present additional complains but the relevance of those in relation to the syndromal background needs validation. Mutations in two enzymes that are required during heparan sulphate synthesis (*EXT1 or EXT2*) are known to cause MO. Previously, we have used zebrafish which harbour mutations in *ext2* as a model for MO and shown that *ext2^−/−^* fish have skeletal defects that resemble those seen in osteochondromas. Here we analyse dental defects present in *ext2^−/−^* fish. Histological analysis reveals that *ext2^−/−^* fish have very severe defects associated with the formation and the morphology of teeth. At 5 days post fertilization 100% of *ext2^−/−^* fish have a single tooth at the end of the 5^th^ pharyngeal arch, whereas wild-type fish develop three teeth, located in the middle of the pharyngeal arch. *ext2^−/−^* teeth have abnormal morphology (they were shorter and thicker than in the WT) and patchy ossification at the tooth base. Deformities such as split crowns and enamel lesions were found in 20% of *ext2^+/−^* adults. The tooth morphology in *ext2^−/−^* was partially rescued by FGF8 administered locally (bead implants). Our findings from zebrafish model were validated in a dental survey that was conducted with assistance of the MHE Research Foundation. The presence of the malformed and/or displaced teeth with abnormal enamel was declared by half of the respondents indicating that MO might indeed be also associated with dental problems.

## Introduction

Multiple osteochondromas (MO), previously known as hereditary multiple exostosis (HME), is a genetic dominant syndrome occurring at the frequency of 1∶50,000 [1] that is caused by mutation of one of the two *EXOSTOSIN* (*EXT*) genes, *EXT1* or *EXT2* [1], [2]. These two *EXT*s encode glycosyltransferases that are crucial for the polymerisation of heparan sulphate (HS). MO manifests itself during the first two decades of life by the development of benign bone tumours (osteochondromas) at multiple sites of the skeleton near growth plates (cartilage structures responsible for elongation of long bones). Osteochondromas predominantly form around the knees and elbows, but all other endochondral bones can be affected. Growing tumours can press on muscles or nerves and thereby cause pain and discomfort. The associations of MO with general pain [3], autism [4], arthritis [5] and scarring [6] have also been reported. MO patients present additional complaints but the relevance of those in the relation to the syndrome requires validation.

Previously, using a zebrafish (*Danio rerio*) mutant called *dackel (dak)* which has a premature stop codon in *ext2*, we established a model for MO [7]. Whereas mice lacking *Ext1* or *Ext2* fail to gastrulate, *ext2^−/−^* fish complete gastrulation and show a strong skeletal phenotype. Although *ext2* has a very broad expression in zebrafish larvae [8], *ext2^−/−^* fish have very specific and consistent phenotypes. We have shown that the cartilage phenotype in *ext2^−/−^* fish resembles that seen in osteochondromas providing evidence that lack of HS proteoglycans (HSPG) affects cartilage morphogenesis, without influencing early cartilage differentiation [7]. We also found that mutation in *ext* genes have a negative effect osteoblast differentiation. In addition we determined that *ext2^−/−^* cells behave autonomously, providing evidence that osteochondroma in humans form as a result of a loss of heterozygosity at an *EXT* locus.

HSPGs are present on membranes and/or in the extracellular matrix in most animal tissues. HSPGs were shown to have very specific spatio-temporal localisation in mice teeth [9]. Similar HSPGs pattern should also be present in human teeth suggesting that alteration of the level or the expression pattern of HS might play a role in dental defects. Indeed, abnormally high levels of HS are know to cause oral defects in patients with mucopolysaccharidoses (MPS), a group of diseases caused by mutations in genes that degrade HS [10]. The presence of dental pathologies was reported by several MO patients, but up to date there is only one case report describing dental abnormalities in a patient with MO and severe vitamin D deficiencies [11]. In order to explore the potential consequences of reduced HS in MO patients we studied the tooth phenotype in *dak* hetero- and homozygote mutants.

Zebrafish teeth are located on the most posterior (5^th^) ceratobranchial arch. The first developing teeth can be observed at 48 hours post fertilisation (hpf). This is the pair of 4V^1^ teeth that by 80 hpf become attached to the middle of the embryonic 5^th^ branchial arch. Development of 4V^1^ is closely followed by the formation of two pairs of neighbouring teeth - 3V^1^ (more medially) and 5V^1^ (more laterally). Both these tooth-pairs are visible by 56 hpf, and early development of 3V^1^ slightly precedes 5V^1^. During later development, 3V^1^ and 5V^1^ become synchronised and both become attached to the arch by 144 hpf [12], [13]. Development and identity of those first three teeth can be followed by analysis of the expression pattern of molecular markers such as *dlx2a*, *dlx2b* and *connexin 43* [12], [14], [15]. Schematic representation of tooth development in the zebrafish larvae is shown in Figure S1. By adulthood, the 5^th^ branchial arch expands to accommodate 11 teeth. These teeth are found in a stereotypical arrangement in adults with the 5 biggest teeth positioned in a ventral row, 4 teeth are a mid-dorsal row and 2 teeth are in the most dorsal row [16]. Throughout juvenile stage and adulthood, old teeth are replaced but the overall organization is maintained.

Tooth development depends on the interplay between epithelium that originates from ectoderm and neural-crest-derived mesenchyme. It is believed that enamel knots (epithelial structures present in mammalian tooth germs) produce signalling molecules that are needed for proper tooth shaping. Enamel knots are absent in zebrafish. However, the expression of molecular markers such as *fgf3* and *fgf4*, that are characteristic for mammalian enamel knots are present in zebrafish larval teeth [15]. Manipulation of fibroblast growth factor (FGF) level was shown influence tooth shape (FGF4; [15]) and tooth number (FGF8; D. Stock from the University of Colorado, USA; personal communication). From mice models it is known that tooth morphogenesis is also regulated by members of the bone morphogenetic protein (BMP) and transforming growth factor (TGF) pathways (more detailed list of genes can be found at http://bite-it.helsinki.fi/). In all these pathways HS plays important role in controlling the ligand-receptor interactions. Hence, a mutation in zebrafish *ext2*, may be expected to affect signalling in teeth by the disruption of signalling events.

Here, we describe a tooth phenotype in *ext2^−/−^* zebrafish and present molecular insights which explain some aspects of the dental defects in fish model for MO. Furthermore, we validate our findings from an animal model by a survey of MO patients. With this work we want to raise awareness among medical doctors, dentists and related professionals of the potential dental problems associated with MO and possibly mucopolysaccharidoses such as Hunter, Hurler, Sanfilippo and Sly syndromes with abnormally high levels of HSPGs.

## Results

### *ext2^−/−^* larvae display a severe tooth phenotype

**Figure 1:**
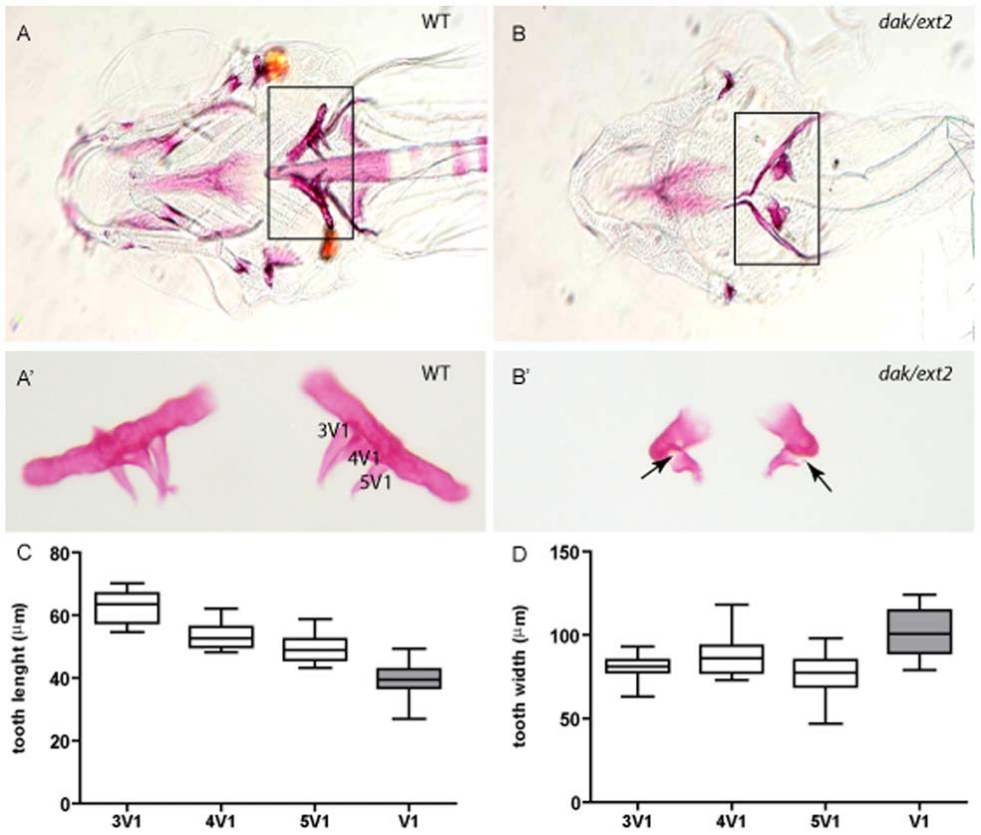
*ext2^−/−^* mutant displays severe tooth phenotype. Ventral view of alizarin-red-stained craniofacial skeleton and teeth at 6 dpf (A, B) and dissected and flat mounted 5^th^ pharyngeal arches with teeth (A′, B′) reveals the presence on each pharyngeal arch of 3 teeth in siblings (A, A′) and only one misshapen tooth in *ext2^−/−^* larvae (B, B′). Note that the rod shaped branchial arch 5 to which the teeth attach is also ossified. Arrows point incomplete ossification of the mutant tooth. Tooth phenotype consisting of one misshapen tooth was observed in all (n>500) analysed *ext2^−/−^* embryos whereas heterozygote fish were indistinguishable from WT. Tooth lengths varies between 3V^1^, 4V^1^ and 5V^1^ in siblings (P<0.003). Each of those teeth was significantly longer then *dak-*tooth (P<0.0001) (C). Tooth widths of 3V^1^ and 5V^1^ were similar between siblings, and both were significantly narrower than 4V^1^ (D). *ext2^−/−^*-tooth was significantly broader than any of the siblings teeth (3V^1^, P<0.0001; 4V^1^, P = 0.023 and 5V^1^, P = 0.0001) (D). White boxes, siblings; grey boxes, homozygote mutant. Scale bar = 0.1 mm.

**Figure 2:**
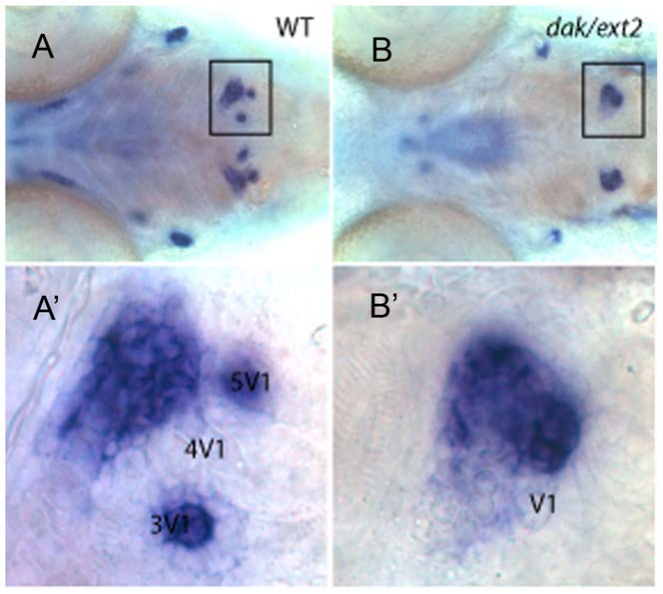
The attachment of the 1^st^ tooth occurs on time in *ext2^−/−^* mutant. The expression of the *osterix* gene at 96 hpf underlines the pharyngeal arches in sibling (A, A′) and *ext2^−/−^* mutant (B, B′). At this stage, *osterix* is also expressed in the sibling in the tooth germs of 3V^1^ and 5V^1^, but lost in the 4V^1^. Mineralised dentine outlines the first to develop and attach – the 4V^1^ tooth. Note that single tooth that does not express *osterix* is also attached into *osterix*-positive pharyngeal arch in the *ext2^−/−^* mutant. A′ and B′ are higher magnification images of A and B. Scale bar = 0.1 mm.

In addition to the previously described cartilage and bone defects in *ext2^−/−^* larvae [7], we now demonstrate that tooth number, development and morphology are also severely affected (Figure 1 and 2). In wild type zebrafish, by 6 dpf, three ossified teeth (3V^1^, 4V^1^ and 5V^1^) attached to each bilateral 5^th^ pharyngeal arch can be visualised by Alizarin red stain (Figure 1A′). All of these teeth have pointed tip and are fully ossified. During the first week of development, *ext2^+/−^* teeth were indistinguishable from WT, whereas in *ext2^−/−^* only one tooth (V^1^) was formed and attached to each 5^th^ arch (Figure 1). Furthermore, *ext2^−/−^*-teeth were always found to be positioned at the end of the 5^th^ arch (Figure 1B′), whereas WT-teeth were located more centrally (Figure 1A′). In all analysed cases (n>20), *ext2^−/−^* teeth were significantly shorter then control teeth (Figure 1C). Width-wise, *ext2^−/−^* tooth was also significantly larger then WT teeth (Figure 1D). Delayed ossification was observed at 4 dpf in *ext2^−/−^* 5^th^ pharyngeal arch and tooth (Figure S2). However tooth attachment occurred on time (Figure 2B′). At 6 dpf, in 90% of *ext2^−/−^* teeth (n = 20), patches of non-ossified areas were found at the base of teeth (Figure 1B′).

**Figure 3:**
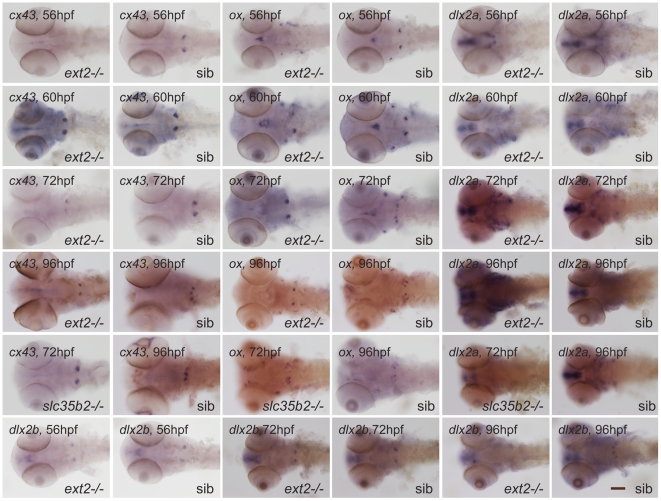
The expression pattern of the dental markers at three stages of development. A panel of markers was used to dissect the identity of the *ext2^−/−^*-tooth (*dak*). Progress of tooth development was monitored at 56, 72 and 96 hpf using *dlx2a*, *dlx2b* and *cx43* markers. *slc35b2^−/−^* (*pic*) mutant was included as it has 2 teeth located to the end of pharyngeal arch. Ossification of teeth in *slc35b2^−/−^* is also delayed and progresses only in one tooth. *ext2^−/−^* and *slc35b2^−/−^* mutants were sorted by phenotype. A, ventral view and B, dorsal view. Scale bar = 0.1 mm.

To further characterise *ext2^−/−^* tooth phenotype we examined tooth development using a set of molecular markers that were described previously in zebrafish [7], [12], [14], [15]. The expression pattern of *dlx2a*, *dlx2b*, *cx43* and *osterix* confirmed the presence of dental defects in the *ext2^−/−^* mutant (Figures 2 and 3). Transcripts of all tested dental markers were detected at 56 hpf in the 4V^1^ teeth in WT and in *ext2^+/−^* larvae. *ext2^−/−^* mutant expressed *dlx2a*, *dlx2b* and *cx43* in one domain at 56 hpf, but the expression level was very weak (Table 1; Figure 3). By 72 hpf transcripts of *dlx2*, *cx43* and *osterix* were detected in all WT and *dak* fish. However, while WT and *dak* siblings clearly expressed *dlx2* and *cx43* at position 4V^1^ and *osterix* at the positions 3V^1^ and 5V^1^, *ext2^−/−^* expressed all markers in one domain (Table 1; Figure 3). By 96 hpf, in all larvae, strong expression of *cx43* and *osterix* were detected in two loci corresponding to 3V^1^ and 5V^1^ in WT and siblings, in one locus in *ext2^−/−^* fish (Table 1; Figure 2 and 3). Transcript of *dlx2b* was detected in all WT and *dak* siblings but not in the homozygote mutant fish. The expression of *dlx2a* was not detected at 96 hpf in any of the fish. We have also analyzed the expression pattern of dental markers in the homozygote *pinscher (pic)* mutant. This strain has a mutation in the *slc35b2* gene and there why lacks sulphation of various molecules including heparan sulphate [7]. *slc35b2^−/−^ larvae* have a similar cartilage phenotype and forms two teeth out of which only one ossifies. We found all markers expressed at 56 and 72 hpf in one locus (Table 1, Figure 3). At later time-point, also weak expression in one locus was detected for *cx43* and *osterix*.

**Table 1:** Summary of the expression pattern of dental markers during early tooth development.

	WT			*dak*			*pic*		
	56 hpf	72 hpf	96 hpf	56 hpf	72 hpf	96 hpf	56 hpf	72 hpf	96 hpf
			3V1^a,b^						
***cx43***	−^b^, 4V1^a^	4V1^a,b^		(V1)^a^	V1^a^	V1^a^	(V1^a^)	(V1^a^)	(V1^a^)
			5V1^a,b^						
***dlx2a***	4V1^a,c,d^	4V1^a,d^	−^a^	(V1)^a^	(V1)^a^	−^a^	(V1)^a^	(V1)^a^	−^a^
		(3v1^d^)	3V1^a,b,d^						
***dlx2b***	4V1^a,c,d^	4V1^a,b,d^	(4v1^a, b, d^)	(V1)^c^	V1^c^	−^a^	V1^a^	na	−^a^
		(5v1^d^)	(5v1^a, b, d^)						
		3V1^a^	3V1^a^						
***osterix***	4V1^a^			(V1)^a^	V1^a^	V1^a^	na	na	(3V1)^a^
		5V1^a^	5V1^a^						

Results from this study (a), were juxtaposed to information available from: Ablooglu *et al*, 2007 [14], (b); Jackman *et al*, 2004 [15], (c); and Borday-Birraux *et al*.,2006 [12], (d). 4V^1^ is the first tooth formed. The development of 4V^1^ is closely followed by the formation of neighbouring teeth. The development of 3V^1^ on the medial side of 4V^1^ slightly precedes the formation of 5V^1^ on the lateral side of 4V^1^. The expression pattern of *dlx2a, dlx2b*, *connexin 43* and *osterix* does not clarify the identity of *ext2^−/−^* tooth. However, comparison of the expression patterns in *ext2^−/−^* and *slc35b2^−/−^* indicate loss of 5V^1^ in both mutants. Week expression is indicated by brackets; -, gene expression was not detected; na, not analysed.

As the tooth development depends on the interplay between epithelial and mesenchymal cells we have also investigated the expression pattern of the *pitx2* gene that is known to be specifically expressed in the zebrafish pharyngeal epithelium [15]. In WT and *dak* mutants at 56 hpf signal was detected in one bilateral domain (Figure S3). The domain in *ext2^−/−^* has similar length to this from siblings, but the patches of pharyngeal epithelium which strongly expressed *pitx2* had width restricted to one cell layer whereas in siblings minimum two cell width was observed (Figure S3).

### Which pathways are impaired in HS-deficient teeth?

**Figure 4:**
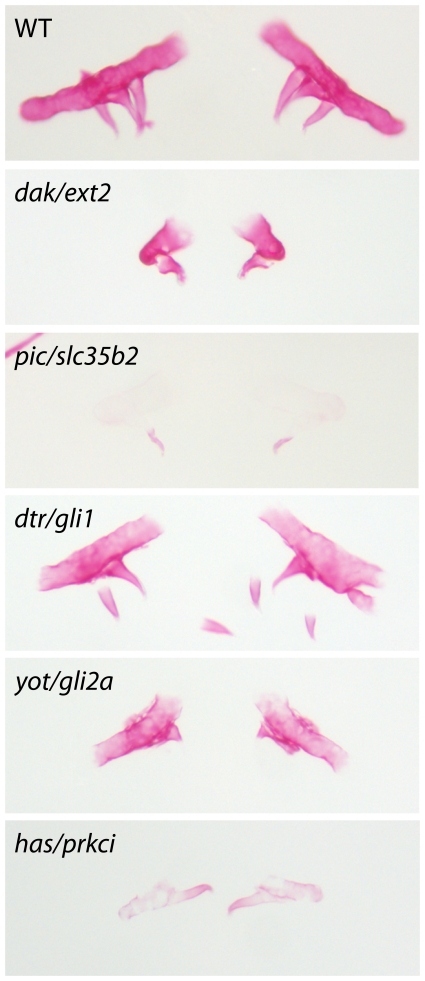
Tooth phenotype in zebrafish mutants affected in FGF, IHH and PKC pathways. Pharyngeal arch together with teeth from 6-days-old WT and *dackel (dak/ext2)*, *detour (dtr/gli1)*, *heart and soul (has/prkci)*, *pinscher (pic/slc35b2) and you too* (*yot/gli2a*) homozygote mutants were stained with alizarin red, dissected and flat mounted. The average length of the 5^th^ arch in WT larvae is 150 µm.

**Figure 5:**
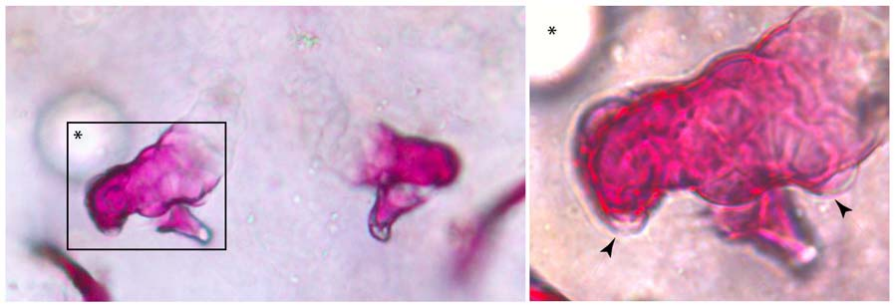
FGF8 stimulates growth of additional tooth-bud-like structures in *ext2^−/−^* mutant. Beads were implanted at 36–39 hpf on one side of the body into an area in between the heart, ear and pectoral fin, where the teeth start to form. At 5 dpf, fish were fixed and stained with Alizarin red. Tooth-buds-like structures were formed on the pharyngeal arch neighboured by FGF-coated bead (arrowhead). Opposite arch was not affected. The tooth-bud-like structures were observed on each side of *ext2^−/−^*-tooth. Asterisk indicate position of the bead.

In order to find out which genetic pathway(s) might be affected in *ext2^−/−^* teeth we screened through known developmental mutants for those that affect tooth number. Out of 18 homozygous mutants, *you too (yot/gli2a)* and *heart and soul (has/prkci)* develop only a single tooth; *acerebellar (ace/fgf8) and boxer (box/extl3)* had 1 to 3 teeth whereas *pinscher (pic/slc35b2)* had two teeth out of which only one ossified (Table 2; Figure 4). In order to verify whether impairment of FGF, PKC and HH signalling indeed affects tooth formation, we have exposed fish to chemical inhibitors of different pathways: SU5402 (FGF), cyclopamine (HH), Gö6976 (PKC), Gö6983 (PKC) and Bimi (PKC). With exception for Bimi that did not cause any visible changes, all other treatments affected tooth formation. Fewer teeth were formed in WT, hetero- and homozygote *dak* mutant fish (Figure S4 for PKC, Figure S5 for FGF, data not shown for IHH). This finding indicated that FGF, IHH and PKC pathways may interact with HS during tooth development. Hence, we decided to test whether activation of those pathways could rescue the *ext2^−/−^*-tooth phenotype. Treatments with purmorphamine (activator of IHH) or PMA (PKC activator) did not have any obvious effect on teeth in the *ext2^−/−^* mutant and its siblings. An example of pharyngeal arches from fish treated with PKC activator is shown in Figure S4. For activation of FGF signalling, we implanted beads soaked in FGF8 into an area in which later forms teeth. Importantly, we have found that local exposure to FGF8 could stimulate tooth-bud development and increase ossification of the 5^th^ arch in mutant larvae (Figure 5).

**Table 2:** Summary of the tooth phenotypes found in various developmental mutants.

mutant name	affected gene	pathway	number of teeth	comments
*acerebellar (ace)*	*fgf8a*	FGF	1–3	fish with more severe phenotype have single tooth
*daedalus (dae)*	*fgf10a*	FGF	3	
*lia (lia)*	*fgf3*	FGF	3	normal shape
*dreumes (dre)*	*sufu*	IHH	3	
*detour (dtr)*	*gli1*	IHH	3	
*you too (yot)*	*gli2a*	IHH	1	broad and short tooth
*u-shaped somites (you)*	*scube2*	IHH	3	
*hi1002*	*casnk1*	WNT/IHH	3	
*pipetail (ppt)*	*wnt5b*	WNT	3	
*silberblick (slb)*	*wnt11*	WNT	3	
*heart and soul (has)*	*prkci*	PKC	1	normal shape
*white tail (mib)*	*mib*	NOTCH	3	normal shape
*boxer (box)*	*extl3*		1–3	3V and especially 5V are delayed
*dackel (dak)*	*ext2*		1	thick and short
*hi307*	*b3gat3*		3	
*hi954*	*uxs1*		3	
*kypek (kny)*	*gpc4*		3	
*pinscher (pic)*	*papst1*		2	only 1 tooth ossifies

Mutant were raised till 6 dpf, fixed and stained with alizarin red. Dissected and flat mounted pharyngeal arches were analysed for number of the attached teeth, number of ossified teeth and abnormalities in tooth shape.

### Tooth phenotype in ext2^+/−^ adult fish

**Figure 6:**
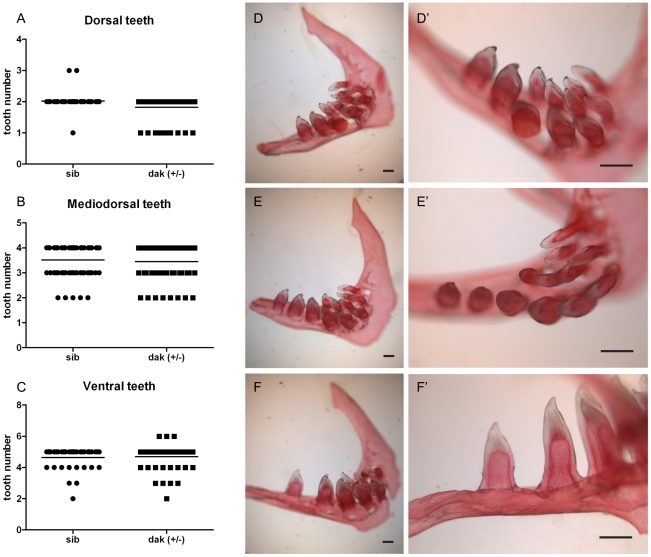
Tooth organization in adult *ext2^(+/−)^* mutant fish. Adult *dak* heterozygote mutant has significantly less teeth in the dorsal row (A); whereas the number of teeth in the mediodorsal row (B) and ventral row (C) are similar to WT. Examples of pharyngeal teeth from adult *ext2^+/−^*: WT-like pharyngeal arch (D, D′), pharyngeal arch with a super-numeral tooth found in the ventral row (E, E′), and large gap between teeth (F, F′). D′, E′ and F′, larger views of D, E and F. Scale bars correspond to 0.1 mm.

**Figure 7:**
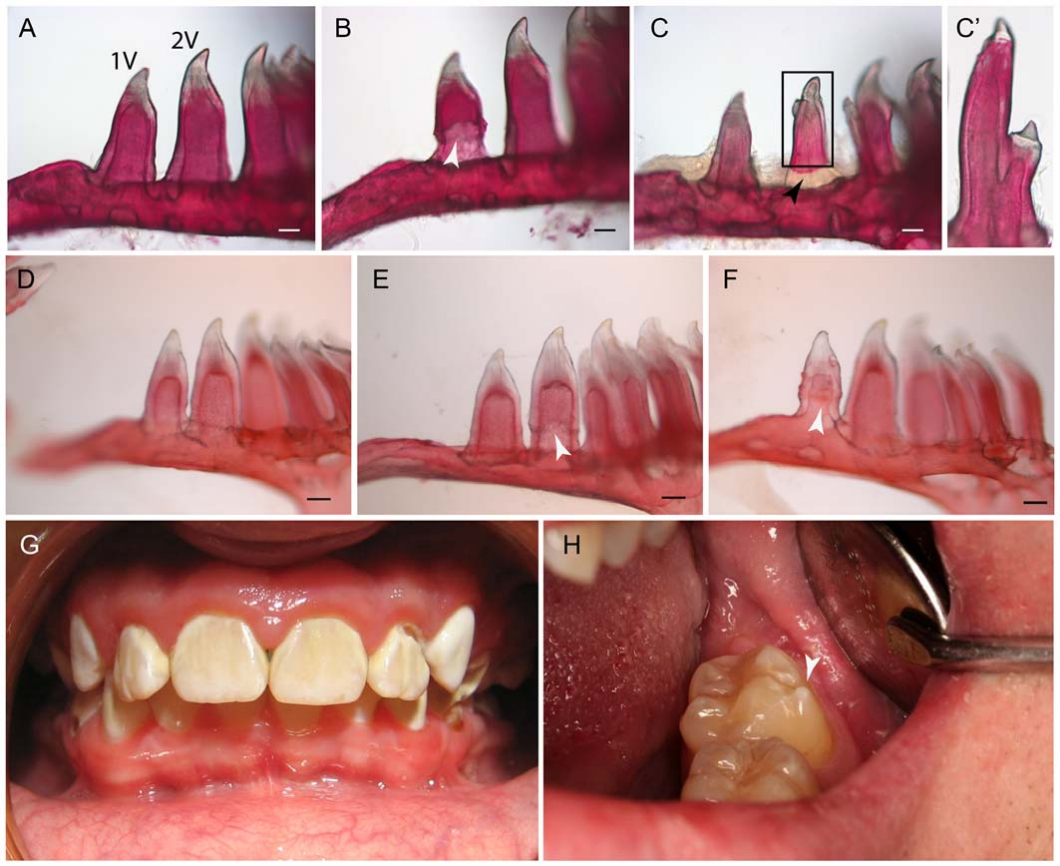
Dental defects are present in 20% of adult *ext2^+/−^* mutant fish. Lateral view of two ventral teeth stained with Alizarin red. In most cases, WT-like teeth were present (A, D). However, on few occasions we also observed: enamel malformation (B, E, F) or misshapen crowns (C, C′). Teeth start to calcify from the tip toward the base; hence the lack of staining at the base of 2V is most likely reflects uncompleted ossification of a recent replaced tooth – see black arrowhead (C). Teeth from MO patients (G, H). Note extra buckle in H (arrow head) which resembles split crown observed in *ext2^−/−^* fish. C′ is a higher magnification of C. White arrows indicate lesions. Scale bars correspond to 0.1 mm.

Since MO patients are heterozygous for mutations in *Ext1* or *Ext2*, we were interested to know if *ext2^+/−^* mutant fish also display tooth defects. No defects were observed in *dak (+/−)* larvae up to 6 dpf (data not shown). A number of tooth pathologies were found in adult fish, and those changes were five times more frequent in heterozygote mutant fish than in the wild type siblings (Figures 6 and 7). As zebrafish teeth are being replaced throughout life, only teeth that were attached to the pharyngeal arch were analyzed. There was no significant difference in the number of teeth number found in WT and mutant fish. However, we have observed changes in the distribution of the teeth (Figure 6). Normally, adult teeth are organised into three rows of teeth: five on the ventral side (1–5V), four in the middle (1–4MD) and two teeth on the dorsal side (1–2D) [16]. In the *ext2^+/−^* mutant, we have found a significant reduction in the number of dorsal teeth, whereas mediodorsal row was unaffected (Figure 6A–C). Interestingly, we have also observed the occurrence of a super-numeral tooth in the ventral row of the *ext2^+/−^* fish (Figure 6C, E, E′) and abnormal gaps between teeth (Figure 6F, F′). Incomplete enamel and split crowns were occasionally found in the *ext2^+/−^* fish, but not in their WT-siblings (Figure 7B–F). Cross section of teeth from adult fish did not show any notable difference in the morphology of *ext2^+/−^* teeth at the microscopic level (Figure S6).

### Does MO affect human teeth?

In order to verify our findings on dental defects in zebrafish model, we have designed a questionnaire (Text S1) in which MO patients and their families were asked to provide information related to their condition and oral health. We have received 23 responses from 22 MO patients and one from a family member that was not diagnosed with MO. 32% of MO patients were genetically tested, out of whom only 3 knew their mutation status and shared this with us (one person had mutation in *EXT1*, one in *EXT2* and one in both genes). The average age of respondents was 39 year-old with minimum age of 5 and maximum age of 68. Among respondents the female to male ratio was 3∶1. 86% of respondents declared that they attended dentists at regular bases (at least once a year). Adult respondents (n = 14) had on average 25.8 teeth out of which 15.7% were sound and untreated. 45% of the MO respondents (n = 22) declared the presence of malformed or displaced teeth and 54% had bleeding gums. 36% of all MO respondents (n = 22) were told by their dentist that they have abnormal enamel and 22% were diagnosed with gingivitis. However, in the population of patients older then 35 year (n = 13) the occurrence of the gum problem was even higher, 61.5% for bleeding gums and 33% for gingivitis. 5% of the respondents often experience toothaches, whereas 38% only occasionally.

## Discussion

MO is known as a genetic syndrome manifesting by the formation of multiple osteochondromas caused by the inactivation of *EXT1* or *EXT2* [17]. All other problems associated with MO, such as bone bowing, short stature or pain are considered as secondary defects caused by growing tumours. However, knowing the importance of HS one could expect that general skeletal, neurological, vascular and other changes would be common in MO. We became interested in the dental issues based upon discussions with members of the MHE Research Foundation (http://mheresearchfoundation.org/HOME.html) and decided to take advantage of the zebrafish model to analyse how reduced HS affects tooth health and development. We used homozygous mutant fish that have a very strong phenotype. Although in this setting, even more subtitle changes are easy to be noticed, the results most likely overemphasises developmental changes caused by the absence of heparan sulphate that will be not observed in patients. This is why, we also analysed adult heterozygous fish. Even though results obtained from zebrafish studies are not directly applicable to patient data, they do give an indication of areas of interest for further study.

### Justification of the zebrafish model for dental studies

Although zebrafish teeth differ from human teeth, they do have a similar organisation (Figure S7). In both cases tooth crowns are made of dentin that is covered with a protective layer of enameloid (fish) or enamel (human); dental pulp that occupies central part of tooth consists of odontoblasts [13], [18]. The pulp cavity contains blood vessels and nerves, but in case of zebrafish this is only true for adult and not for larval teeth [19]. Furthermore, in contrast to human teeth that have roots, the presence of cementum was not described in zebrafish. This might be due to the fact that zebrafish are polyphyodont and hence they do not have permanent teeth as mammals do.

### Why does only one tooth forms in dak homozygote mutants and what is its identity?

Comparing the expression patterns of various dental markers in the *ext2^−/−^* mutant that has one tooth, *slc35b2^−/−^* mutant that has 2 teeth and WT with three teeth, 5V^1^ tooth as a possible identity of the single *ext2^−/−^* tooth can be ruled out as no expression of dental markers was seen in any of the mutants. However, our data are no conclusive for other tooth identities. It might be that the tooth is 4V^1^ that is delayed or 3V^1^ that develops faster. Alternatively, *ext2^−/−^* tooth might result from V^1^ 3 and 4 being fused together. In mammals, tooth development depends on the interplay between epithelium that originates from ectoderm and neural-crest-derived mesenchyme. Despite the disagreement whether zebrafish epithelium originates for ectoderm or endoderm, tooth development in *ext2^−/−^* might be caused by partial loss of contact between epithelium and mesenchyme. In fact, we have found that *pitx2*-expression domains in the pharyngeal epithelium do not undergo thickening in *ext2^−/−^* (Figure S3). In contrast to the rod-like shape of the pharyngeal cartilages in WT, *ext2^−/−^* pharyngeal are shorter and thicker [7], [20]. Moreover, abnormal accumulation of cells undergoing apoptosis was noted at the lateral end of the *ext2^−/−^* pharyngeal (Figure S8). Hence, it is possible that 5^th^ arch is not long enough to interact with the lateral part of epithelium i.e. the region where 5V^1^, and possibly 4V^1^, should be formed. This hypothesis could be strengthened by the fact that *ext2^−/−^* tooth is formed at the very distal end whereas the WT-teeth are always located to the middle of the pharyngeal arch. Furthermore, in the *slc35b^−/−^* mutant, a similar tooth loss coincides with shortening of the pharyngeal arch (Figure 4). Thus, defects in craniofacial morphogenesis may explain some aspects of the tooth phenotype.

The partial gaps in the ossification of the *ext2^−/−^* tooth at 6 dpf could indicate signs of natural events prior to tooth replacement. However, since *ext2^−/−^* tooth attaches on time and patches of non-ossified areas were also found in *ext2^−/−^* teeth already at 4 dpf, hence it is more likely that incomplete ossification reflect pathological changes in *ext2^−/−^* teeth. Given the osteoblast differentiation is reduced in *ext2^−/−^* fish [7], it is possible that also similar defects affect odontoblast and this incomplete ossification results from diminished odontoblast numbers.

### Why tooth phenotype is observed only in every fifth heterozygote dak mutant?

In adult zebrafish, dental changes were observed only in 20% of fish that are known to carry a mutation in the *ext2* gene. Which raises the question why the remaining 80% of fish heterozygous for a mutation in the *ext2* gene remain asymptomatic? Considering the huge variability of the bone phenotype in MO, involvement of other gene(s) cannot be excluded. However, the occurrence of the loss of heterozygosity (LOH) in tumours was confirmed in a number of solitary and multiple osteochondromas [21]–[23], which suggests that dental defects might take place as a result of a second hit mutation. Hence, if dental defects arise from a LOH, they would be sporadic and heterogeneous in nature. Moreover, the dental changes that we found in the *ext2* heterozygote mutant fish, were not specific. Similar changes could be observed in wild type fish, although at lower frequency. This is why they may have been previously overlooked.

### Validation of the finding from the zebrafish model for MO

Mouse models have also been established for MO and used for studies on the formation of tumours [24]–[28] and synovial joints [29]. Although none of the authors characterised teeth of the *Ext1* or *Ext2-*mutant mice, dental defect were described in *Ndst1-*null mice [30]. NDST1 is an enzyme that acts downstream of EXTs in the biosynthesis of heparan sulphate.

Online surveys might be biased towards a group having an active interest in given topic. Although, two-thirds of our respondents reported the presence of dental problems, the results of our dental survey still should be used as a guideline only. As survey was returned by one non-MO respondent only, we used general national statistic as an indicator of the frequencies of dental defects in normal population. According to a survey carried in the UK in 1998, adult dentate has on average 24.8 teeth with 15.3% of sound and untreated teeth (Office for National Statistics, UK). Although the number of teeth in adult human population varies depending on age, gender and country, in comparison to the UK population, our MO patient do not present any deviation form the norm, neither in the total number of teeth nor the number of untreated and good teeth. This is in line with our observation from zebrafish heterozygote adults carrying mutation in the *ext2* gene that showed no significant difference in the tooth number as compared to WT siblings. The presence of misplaced and/or malformed teeth found from this zebrafish study was also indicated by 45% of the MO patients. Every third MO patient stated that was told by a dentist that they have abnormal (“too thin”) enamel. Unfortunately, technical limitations did not allow us to test enamel thickness in our zebrafish model.

In addition to tooth defects observed in 100% of homozygote mutant larvae and in 20% of heterozygote adult fish, the dental questionnaire brought a high percentage of MO patients having problems with bleeding gums and gingivitis to light. However, as the occurrence of bleeding gums and/or gum inflammation are very frequent in normal population and the number of respondents were low (n = 23) we can not conclude if there are any significant differences between those two groups. It should be noted, that dental defects observed in MO patients do not look specific, and hence a special screening programme would be needed in order to tell whether tooth lesions, plaques on teeth and gingivitis are due to the impropriate dental hygiene or indeed due to MO.

### How many people might be affected?

The frequency of MO occurrence was estimated at 1∶50000. However since a) some of the osteochondromas are asymptomatic and b) genotyping or full body x-arrays or MRI are not routinely done even on patients who are diagnosed with solitary osteochondroma than it is possible that many people are undiagnosed or misdiagnosed and the over all number of people having MO might be substantially higher. Recently Yasuda and colleagues [30] described dental defect in mice with null mutation in the *Ndst1* gene. This implies that putative patients with a mutation in other genes involved in the biosynthesis of HS might increase the number of people with dental defects due to HS insufficiency. However, as patients with mucopolysaccharidosis who accumulate abnormally high levels of HS also have dental defects, it seems that any imbalance in HS might result in dental defects.

Here, we demonstrated that mutation in *ext2* causes dental defect in zebrafish. Furthermore, we validated this observation in a pilot study on a group of MO patients. Our findings strongly suggest that MO is associated with dental problems. Problems with teeth might severely affect not only the self-esteem but also health of patients, hence it is important that MO patients receive appropriate dental care. More detailed studies on a larger group of patients should be perform for better understanding the clinical spectrum of this novel aspect of MO.

## Materials and Methods

### Animals and histology

Unless stated otherwise, all methods were based upon standardized protocols [31]. Zebrafish (*Danio rerio* H.) TL strain was used as wild type (WT). Homozygote *acerebellar (ace, fgf8a^ti282c^)*, *boxer (box, extl3^tw24^)*, *dackel (dak, ext2^to273b^)*, *daedalus (dae, fgf10a^tbvbo^)*, *detour (dtr, gli1^tm276b^)*, *dreumes (dre, sufu^tm146d^)*, *lia (fgf3, lia^H0006-01^)*, *heart and soul (has, prkci^m129^)*, *knypek (kny, gpc^V348^)*, *pinscher (pic, slc35b2^14MX^)*, *pipetail (ppt, wnt5b^ti265^)*, *hi307(β3gat3^hi307^)*, *hi954(uxs1^hi954^)*, *hi1002 (csnk1α1^hi1002^)*, *silberblick (slb, wnt11^tx226^)*, *u-shaped somites (you, scube^ty97^)*, *you too* (*yot, gli2a* ^ty119^) and *white tail (mib^ta52b^)* mutants were obtain in obtained in natural crosses and staged according to Kimmel *et al.* [32] Cartilages and bones were stained with Alcian blue and alizarin red respectively as described in [7].

### Ethical statement

Patients data were obtained and handled according to ethical guidelines as described in the Code for Proper Secondary Use of Human Tissue in the Netherlands of the Dutch Federation of Medical Scientific Societies (www.federa.org). The institutional Review Board at the Department of Pathology at Leiden University Medical Centre imposes the use of the guidelines to any study performed with the use of human material. As the material was used in an anonymous, coded form informed consent was not required. Zebrafish work was conducted in the Netherlands accordingly to Dutch law (article 9, Experiments on Animals Act) and did not require any licence for early stages. Studies performed with zebrafish in the UK were performed with Home Office approval under license PPL 40/2919.

### *In situ* hybridization

Whole mount *in situ* hybridisation was performed as described in [33]. Antisense probes were amplified with M13 primers using *dlx2a*, *dlx2b*, *pitx2* [34], *cx43* [14] and *osterix* [35] plasmids as templates. Goat anti-DIG fab fragments (Roche) and NBT/BCIP substrate (Sigma) were used to develop the *in situ*. Prior to photo documentation embryos were cleared and preserved in 75% glycerol.

### Measurements

Teeth were analysed in homozygote mutants and WT at 6 dpf. Measurements of the tooth length and width were taken on pictures of the dissected and flat mounted pharyngeal arches. Teeth were also dissected out along with whole pharyngeal arches from one year old fish. As zebrafish teeth undergo replacement throughout life, only attached teeth were analysed in this study. Teeth organisation was examined in adult heterozygote mutants and their siblings and compared to the pattern described previously [16]. Teeth from adult fish were also screened for other abnormalities such as incomplete enamel formation and crown splits. Teeth from WT and *ext2^+/−^* fish were cross-sectioned and subjected to histological analysis. Groups of minimum ten fish were used for each measurement.

### Chemical treatment

Drugs were added into Petri dish with 50 manually dechorionated embryos in 25 ml E3. FGF inhibitor (SU5402), IHH inhibitor (cyclopamine), IHH activator (purmorphamine), PKC inhibitors (Gö6976, Gö6983 and Bimi) and PKC activator (PMA) were all purchased from Merck/Calbiochem. A 10 mM stock solution of cyclopamine in Ethanol was added into fish water to a give final concentration corresponding to 10–100 µM. Stock solution of 40 µM SU5402 in DMSO was diluted in fish water up to final concentration of 40–200 nM. 10 mM stocks of Gö6976, Gö6983 and Bimi in DMSO were added into fish water and used at final 10–100 µM concentration. 1 mM PMA stock in DMSO was diluted in fish water and tested at concentrations: 0.02–1 µM. Tested compounds and corresponding solvents (controls) were added to E3 at 24, 36 and 50 hpf and either kept for 24 h and washed off with fresh E3 or left unmoved up to 6 dpf, when fish were fixed and subjected to Alcian blue and alizarin red staining.

### Bead implants

Heparin beads (Sigma) were coated with mouse recombinant FGF-8b (R&D systems) as described [36], [37]. BSA-coated beads were used for control. Beads were implanted at 36–39 hpf into an area in between the heart, ear and pectoral fin, where the teeth start to form. Fish were raised until 96 hpf for molecular analysis (mRNA *in situ* hybridisation) and until 5 dpf for histological examination (alizarin red stain). Mice and zebrafish FGF8 share a high level of conservation (Figure S9).

### Dental questionnaire

Dental questionnaire was design in a way that it could be answer without the need of consulting a dentist or another medical professional. The questionnaire was intended as an online survey for patients and their families (socio-economically matched control). The survey was placed on the EuroBoNet homepage (http://www.eurobonet.eu/news/News.php) and at the MHE Research Foundation website (http://mheresearchfoundation.org/HOME.html) located on an encrypted secure webpages. In addition, three patient support groups were directly invited to participate in the survey. Responses were collected, coded by an independent body in order to keep anonymity and analysed. The complete questionnaire is given in the Text S1.

## Supporting Information

Figure S1 **Simplified representation of the tooth development in the zebrafish larvae.** During the first week of life, zebrafish develops pharyngeal teeth at three positions only, 3V, 4V and 5V. Tooth 4V^1^ is the first tooth to differentiate (48 hpf), attach into the pharyngeal arch (80 hpf) and undergo replacement by 4V^2^ at 12 days post fertilisation. Teeth, 3V and 5V start to differentiate at 56 hpf and become attached at 144 hpf [12], [13].
(TIF)

Figure S2 **Tooth ossification is delayed in** ***ext2^−/−^*** **mutant.** Is indicated by Alizarin red stain at 96 hpf, single tooth is ossified in both *ext2^−/−^* mutant and its siblings. Interestingly, the ossification of the pharyngeal arches starts in the mid part in siblings (A, A′) and at the end of arch in *ext2^−/−^* mutant (B, B′). Moreover, weaker intensity of the Alizarin red in *ext2^−/−^* suggests general delay in ossification. A′ and B′, line outline of the branchial arch 5 and attached teeth. Scale bar = 0.1 mm.
(TIF)

Figure S3 **The expression pattern of** ***pitx2*** **indicates slight defect in thickening of the pharyngeal epithelium in the** ***ext2^−/−^*** **mutant.** *pitx2*-expressing bilateral domain in the *ext2^−/−^* are of similar length but more narrower than the one from siblings. A, siblings and B, *ext2^−/−^* at 56 hpf. A′ and B′, magnification of the pharyngeal area. Scale bar = 0.1 mm.
(TIF)

Figure S4 **Inhibition of PKC affects tooth formation.** Similarly to *has* (PKC) mutant, one tooth-phenotype was also observed in fish treated with PKC inhibitor. PMA – activator of PKC does not stimulate formation of additional teeth in WT, nor rescues tooth phenotype in the *dak* homozygote mutant. Cartilaginous skeletons were stained with Alcian blue at 6 dpf. Pharyngeal arches were dissected out and flat mounted.
(TIF)

Figure S5 **Inhibition of FGF by SU5402 tooth formation in WT and** ***dak*** **mutant.** Embryos were treated from 50 hpf till 6 dpf. The *ext2^−/−^* fish treated with SU5402 does not form teeth hence picture was not included. Alizarin-red-stained pharyngeal arches were dissected and flat mounted. Arrow points a single bilateral tooth formed in *dak* siblings.
(TIF)

Figure S6 **Tooth morphology in adult** ***dak*** **heterozygote mutant.** Cross section of teeth from adult fish did not reveal any obvious morphological differences between WT and *ext2^−/−^*. 4 µm sections of teeth were stained with haematoxylin and eosin.
(TIF)

Figure S7 **Schematic representation of adult human and zebrafish teeth.** Organisation of the zebrafish tooth was adapted from work by Neues and colleagues [18].
(TIF)

Figure S8 **Accumulation of cells undergoing cell death at the end of the pharyngeal arch in the** ***ext2^−/−^*** **mutant.** TUNEL staining was performed in fish at 6 dpf. Pharyngeal arches were dissected and flat mounted.
(TIF)

Figure S9 **Alignment of the zebrafish and mouse FGF8 shows high level of conservation between proteins.** Accession numbers: mouse FGFb, P37237-2; zebrafish FGF8a, Q5PRC3; and zebrafish FGF8b, B3DJ36.
(TIF)

Text S1 **Dental questionnaire for MO patients and their families.**
(PDF)
