# Supplementary figures and images for: HSPG-Deficient Zebrafish Uncovers Dental Aspect of Multiple Osteochondromas
Source: PLoS One. 2012 Jan 11;7(1):e29734. doi: 10.1371/journal.pone.0029734 (PMC3256178; doi:10.1371/journal.pone.0029734)

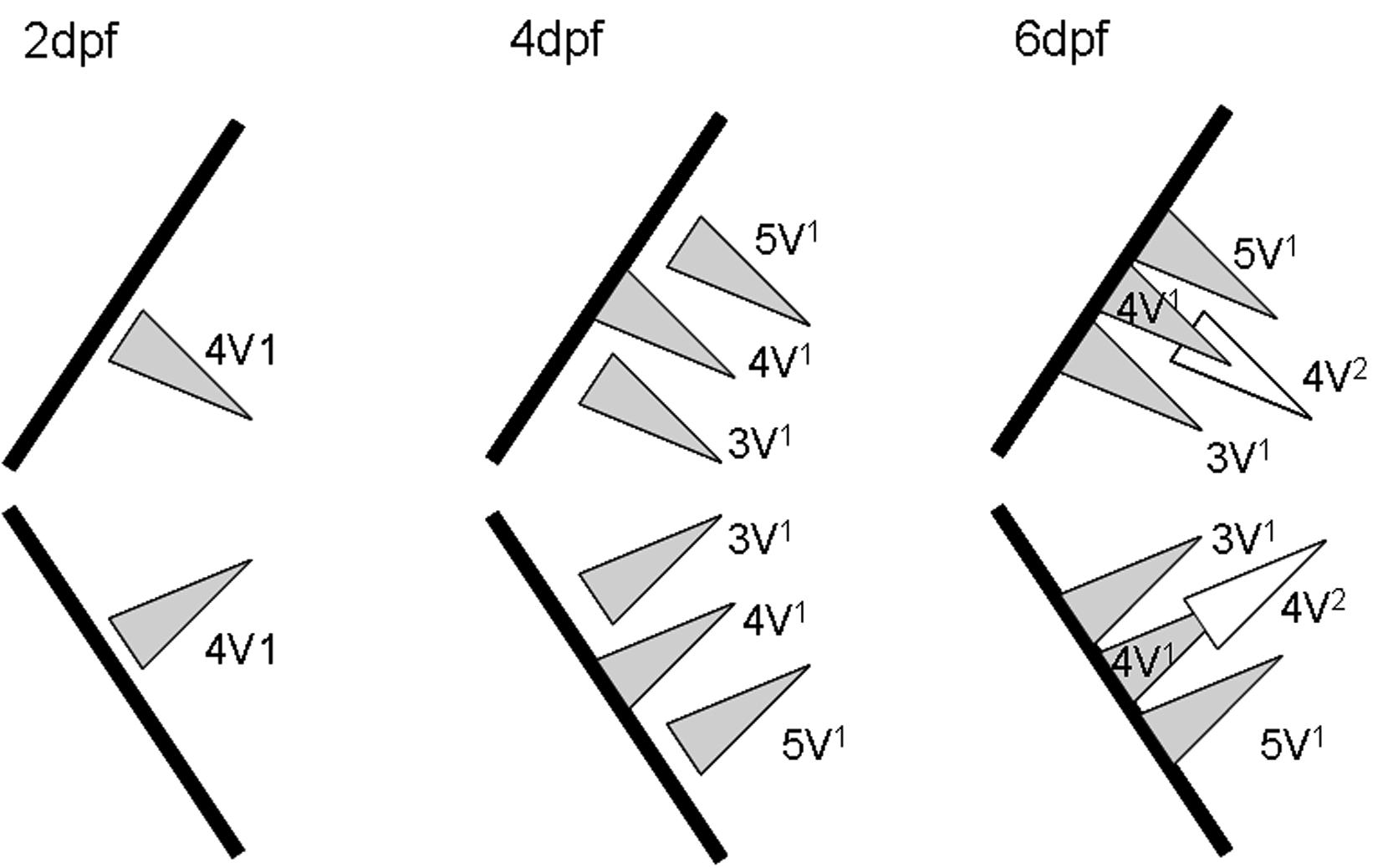

Supplement: Figure S1 — Simplified representation of the tooth development in the zebrafish larvae. During the first week of life, zebrafish develops pharyngeal teeth at three positions only, 3V, 4V and 5V. Tooth 4V1 is the first tooth to differentiate (48 hpf), attach into the pharyngeal arch (80 hpf) and undergo replacement by 4V2 at 12 days post fertilisation. Teeth, 3V and 5V start to differentiate at 56 hpf and become attached at 144 hpf [12], [13]. (TIF) [file pone.0029734.s001.tif]

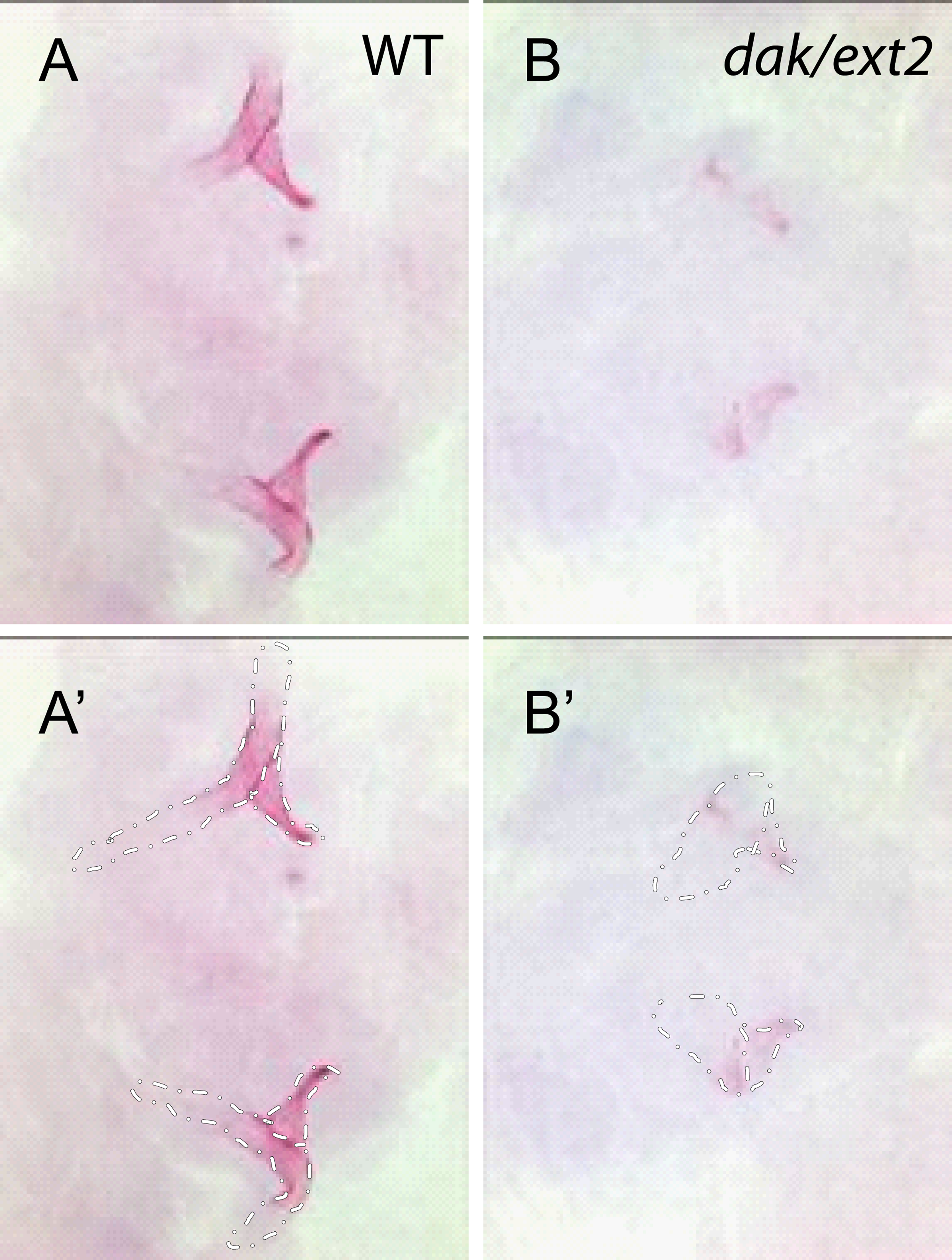

Supplement: Figure S2 — Tooth ossification is delayed in ext2−/− mutant. Is indicated by Alizarin red stain at 96 hpf, single tooth is ossified in both ext2−/− mutant and its siblings. Interestingly, the ossification of the pharyngeal arches starts in the mid part in siblings (A, A′) and at the end of arch in ext2−/− mutant (B, B′). Moreover, weaker intensity of the Alizarin red in ext2−/− suggests general delay in ossification. A′ and B′, line outline of the branchial arch 5 and attached teeth. Scale bar = 0.1 mm. (TIF) [file pone.0029734.s002.tif]

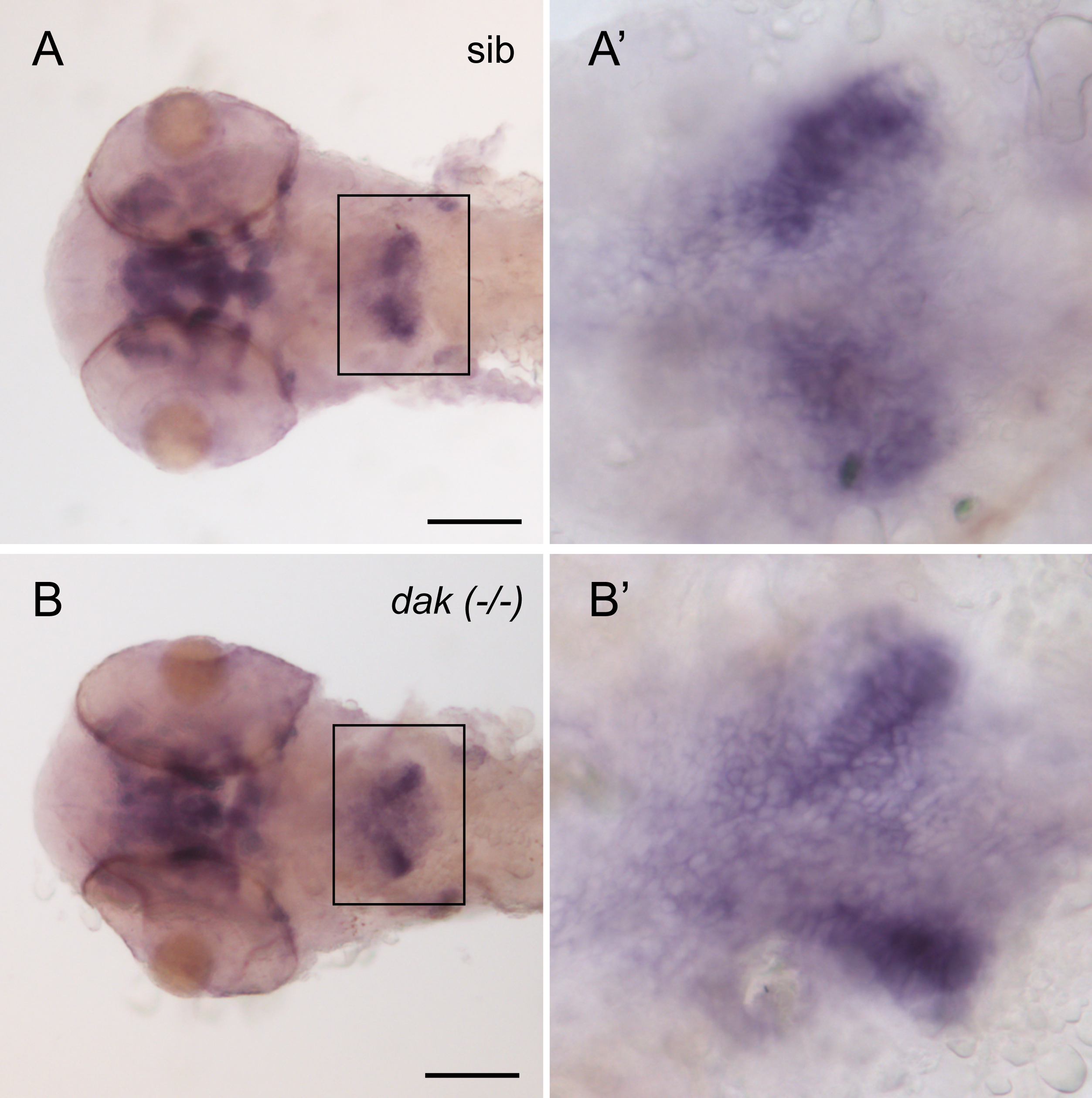

Supplement: Figure S3 — The expression pattern of pitx2 indicates slight defect in thickening of the pharyngeal epithelium in the ext2−/− mutant. pitx2-expressing bilateral domain in the ext2−/− are of similar length but more narrower than the one from siblings. A, siblings and B, ext2−/− at 56 hpf. A′ and B′, magnification of the pharyngeal area. Scale bar = 0.1 mm. (TIF) [file pone.0029734.s003.tif]

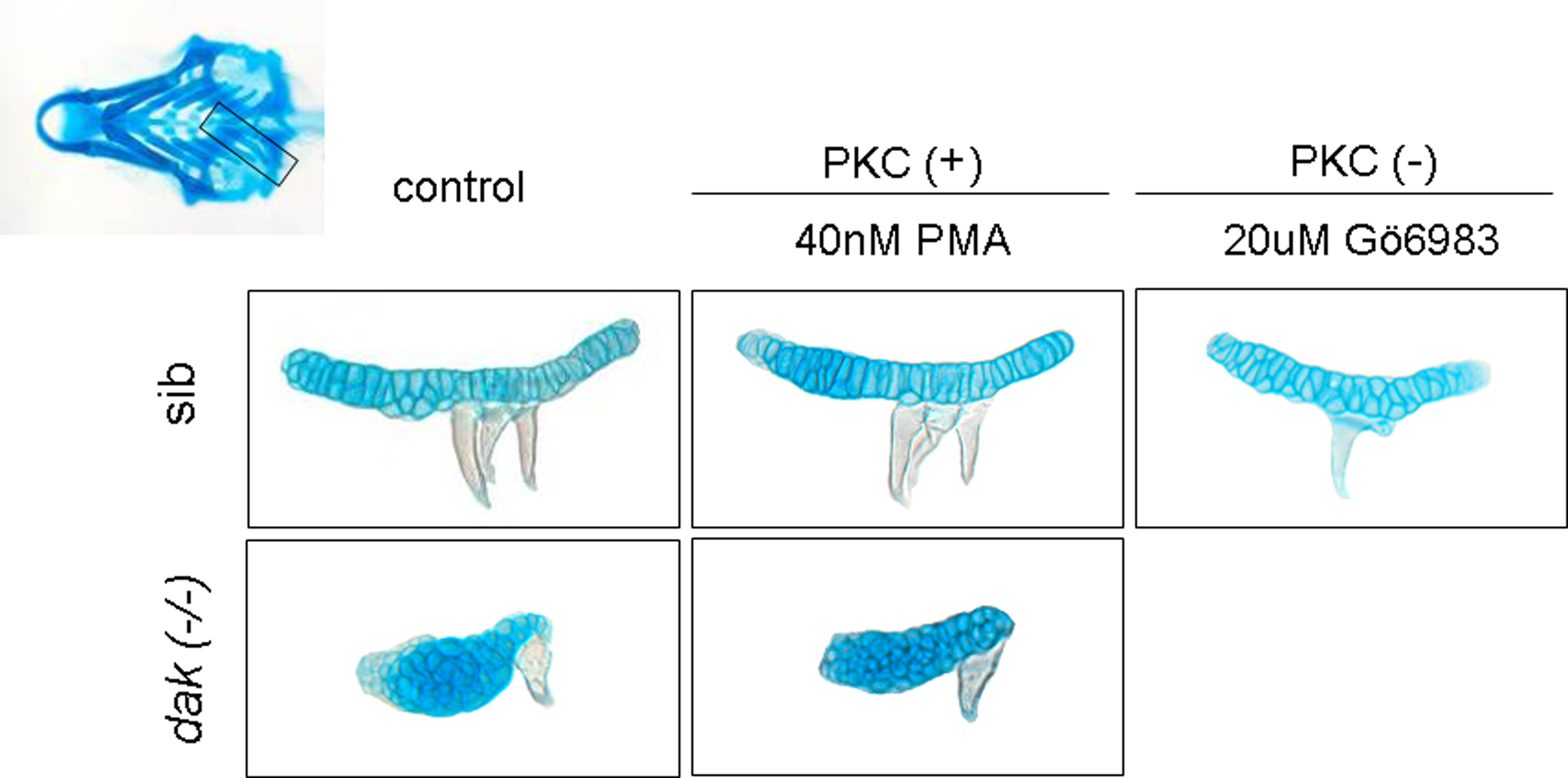

Supplement: Figure S4 — Inhibition of PKC affects tooth formation. Similarly to has (PKC) mutant, one tooth-phenotype was also observed in fish treated with PKC inhibitor. PMA – activator of PKC does not stimulate formation of additional teeth in WT, nor rescues tooth phenotype in the dak homozygote mutant. Cartilaginous skeletons were stained with Alcian blue at 6 dpf. Pharyngeal arches were dissected out and flat mounted. (TIF) [file pone.0029734.s004.tif]

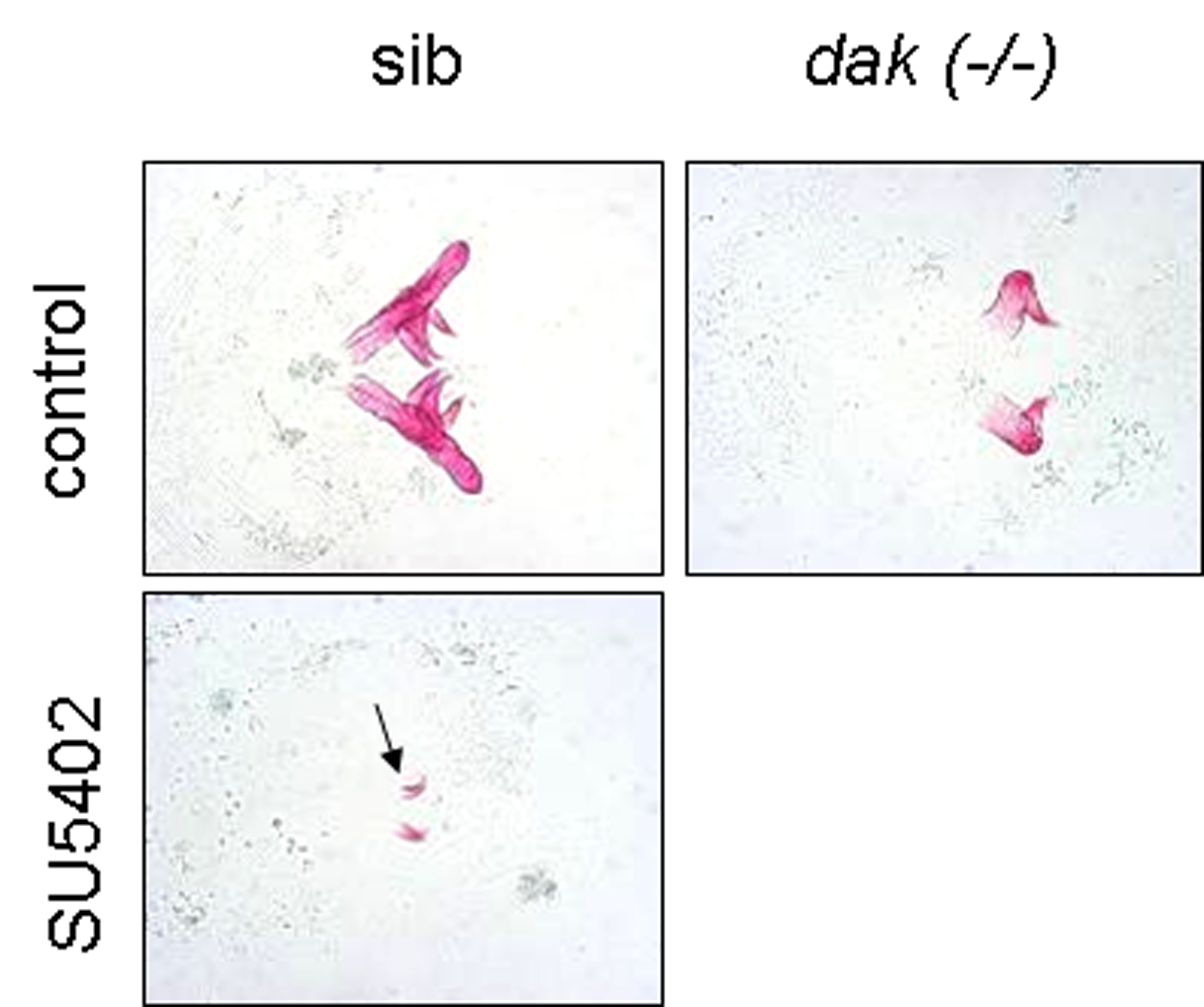

Supplement: Figure S5 — Inhibition of FGF by SU5402 tooth formation in WT and dak mutant. Embryos were treated from 50 hpf till 6 dpf. The ext2−/− fish treated with SU5402 does not form teeth hence picture was not included. Alizarin-red-stained pharyngeal arches were dissected and flat mounted. Arrow points a single bilateral tooth formed in dak siblings. (TIF) [file pone.0029734.s005.tif]

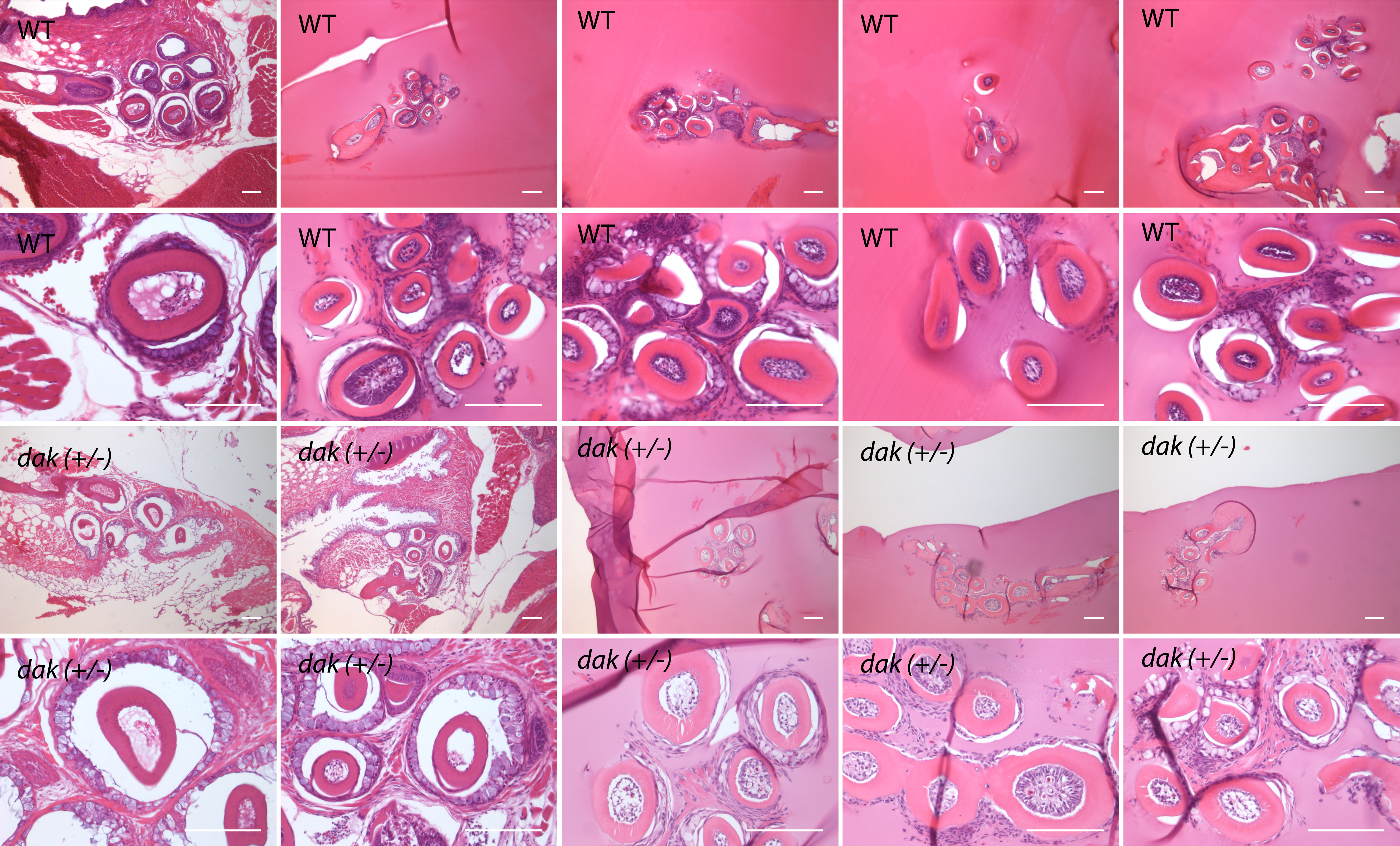

Supplement: Figure S6 — Tooth morphology in adult dak heterozygote mutant. Cross section of teeth from adult fish did not reveal any obvious morphological differences between WT and ext2−/−. 4 µm sections of teeth were stained with haematoxylin and eosin. (TIF) [file pone.0029734.s006.tif]

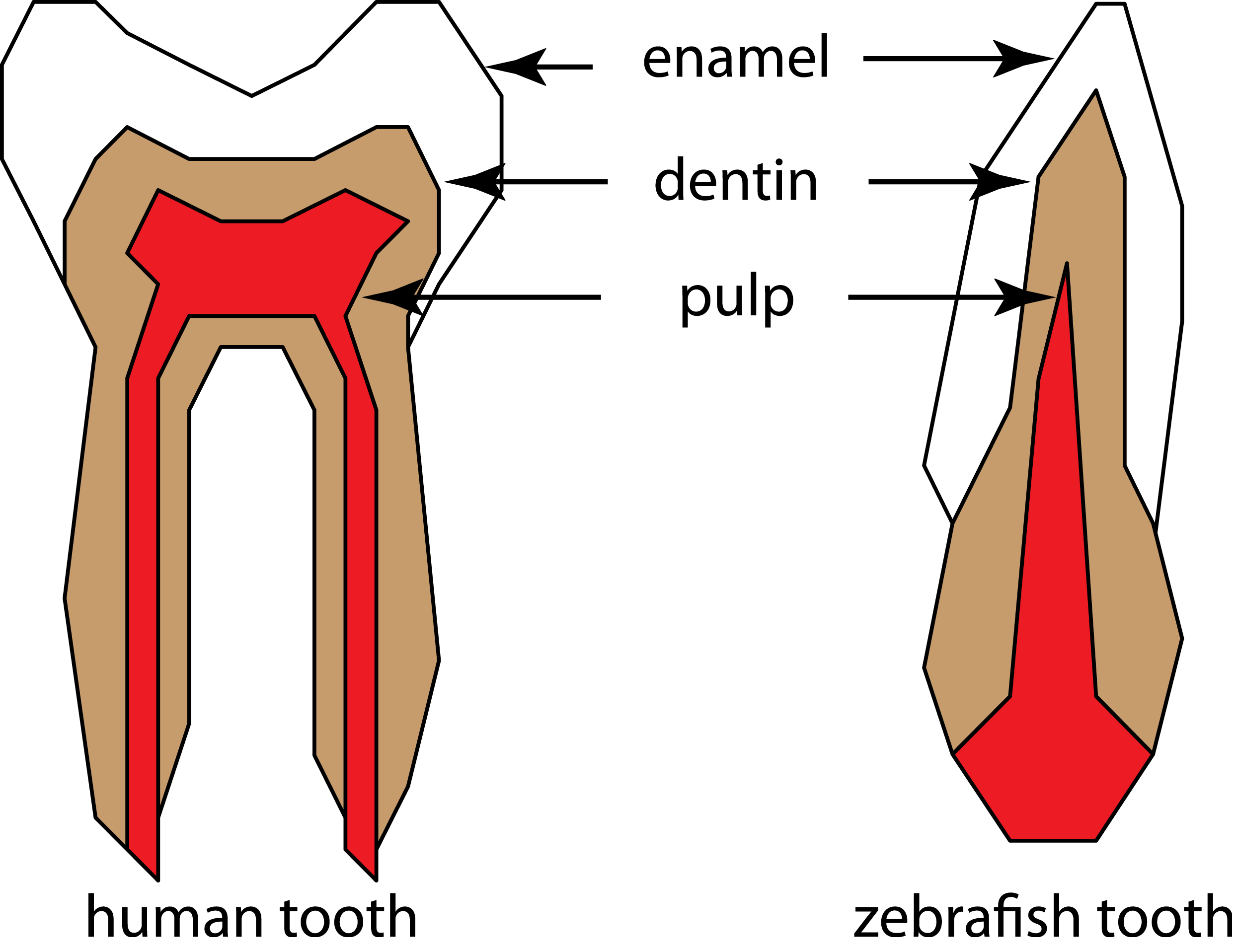

Supplement: Figure S7 — Schematic representation of adult human and zebrafish teeth. Organisation of the zebrafish tooth was adapted from work by Neues and colleagues [18]. (TIF) [file pone.0029734.s007.tif]

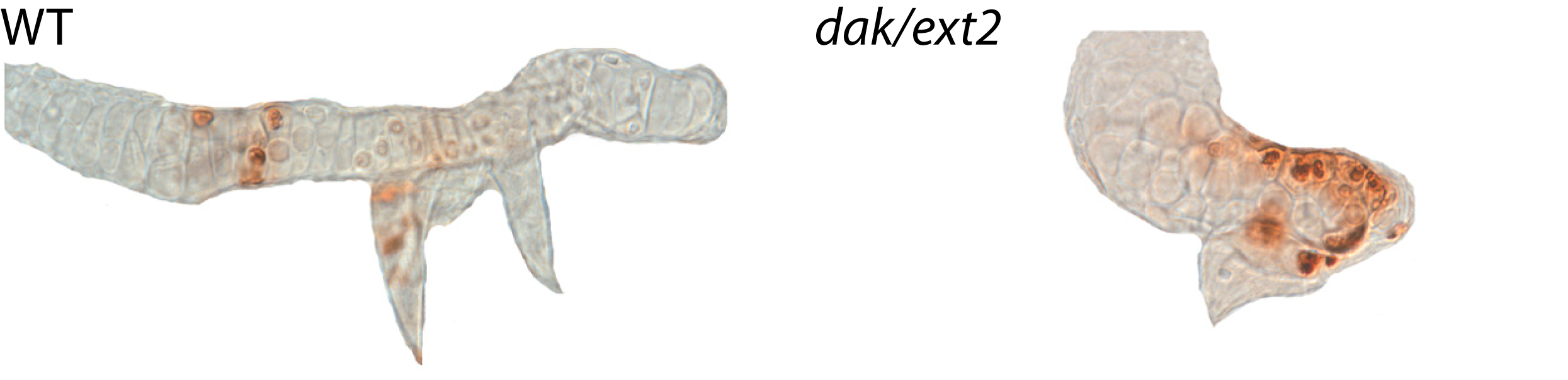

Supplement: Figure S8 — Accumulation of cells undergoing cell death at the end of the pharyngeal arch in the ext2−/− mutant. TUNEL staining was performed in fish at 6 dpf. Pharyngeal arches were dissected and flat mounted. (TIF) [file pone.0029734.s008.tif]

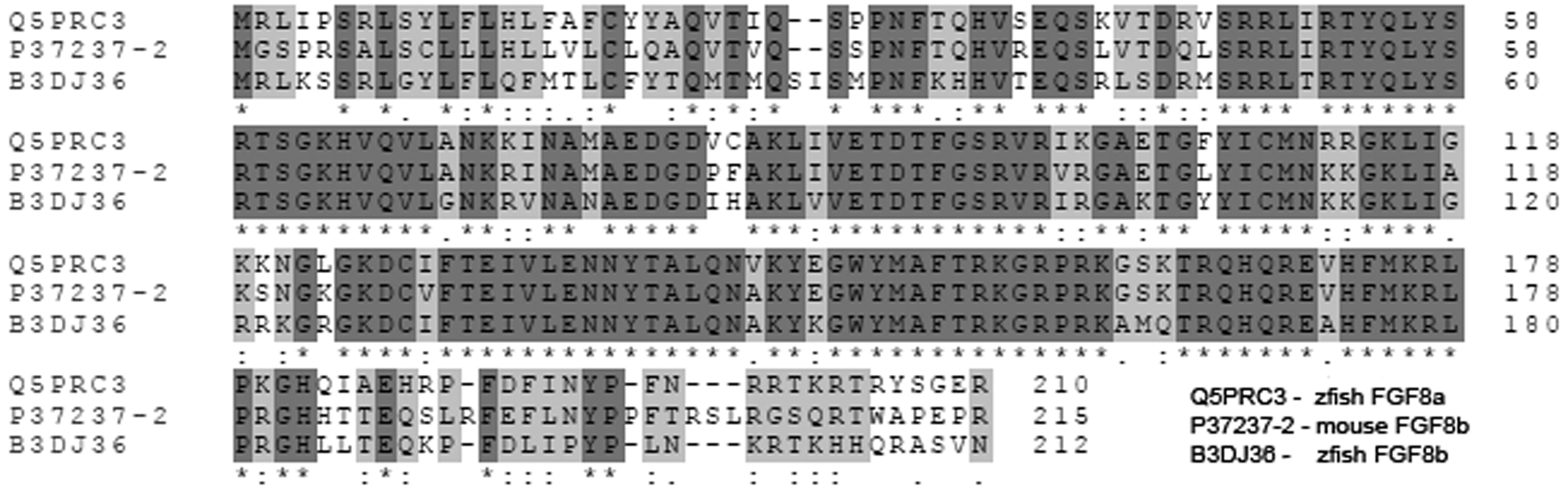

Supplement: Figure S9 — Alignment of the zebrafish and mouse FGF8 shows high level of conservation between proteins. Accession numbers: mouse FGFb, P37237-2; zebrafish FGF8a, Q5PRC3; and zebrafish FGF8b, B3DJ36. (TIF) [file pone.0029734.s009.tif]
