# Supplementary material for: HSPG-Deficient Zebrafish Uncovers Dental Aspect of Multiple Osteochondromas
Source: PLoS One. 2012 Jan 11;7(1):e29734. doi: 10.1371/journal.pone.0029734 (PMC3256178; doi:10.1371/journal.pone.0029734)
Supplement: Text S1 — Dental questionnaire for MO patients and their families. (PDF) [file pone.0029734.s010.pdf]

Multiple Osteochondromas (MO) previously known as Hereditary Multiple Exostoses (HME) or Multiple Hereditary Exostoses (MHE) is relatively rare disorder associated with formation of bone outgrowths at multiple sites in the skeleton. The presence of dental problems has been communicated to us by some MO patients, but as far as we know, there are no medical data published that would confirm this observation. As problems with teeth might severely affect not only the self esteem but also general health, hence we recognize that dental health in MO patients needs to be investigated.

With the support and collaboration of Pancras Hogendoorn, M.D., Ph.D. and Malgorzata Wiweger, Ph.D. from the Leiden University Medical Center(LUMC) The Netherlands a questionnaire has been designed that could be answered without need of seeing any dentist or other medical professionals. We hope that this survey will contribute to better understanding of the MO disorder and improve the quality of care given to MO patients.

All Support groups and individuals who suffer from MO are invited to participate in this study by answering the questionnaire. In order to reach as many MO patients as possible and still be able to manage the project researchers from LUMC decided to place an on-line survey on the MHE Research Foundation website located on an encrypted secure webpage. All responses will be collected by Mrs. Sarah Ziegler (Vice President of the MHE Research Foundation, National Director of Research & Coordinator of Clinical Information, Executive Director of the National MHE Research Registry), encoded in order to keep anonymity /confidentiality of the respondents and forwarded to the team of researchers at the LUMC for analyses. The research team aims to make the results publicly available in a form of an article published in a scientific journal, so basic researchers; medical professionals and people affected by MO will be able to read the results.
As a quick reference a picture of the teeth has been placed on this page.

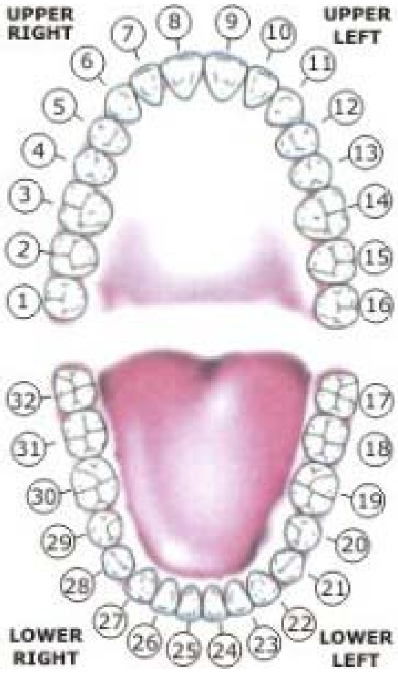

Please click and fill in the answers to all questions that apply in your case.

1. Date of birth, Year ☐ Month ☐ Day ☐

Have you started puberty? No ☐ Yes ☐ (Please answer this question if the respondent is under the age of 21 years old)

2. Do you have a family history of MO on your:

Mother side? No ☐ Yes ☐

Father side? No ☐ Yes ☐

3. Do you have any brothers or sisters who have been diagnosed with MO? If yes please fill in answer.

☐

4. Were you diagnosed with MO or any other term used for this disease? No ☐ Yes ☐

5. Have you been genetically tested for MO? No ☐ Yes ☐

If you have been genetically tested for MO, please click the answer that describes you situation:

NO mutation was found? No ☐ Yes ☐

A mutation in the *EXT1* gene was found No ☐ Yes ☐

A mutation in the *EXT2* gene was found No ☐ Yes ☐

I do not know the results of my genetic test. No ☐ Yes ☐

6. If your mutation was found and you know detail about its type and location please give details (this information would be given in the genetic testing report).

☐

7. How do you consider yourself affected by MO?

Severely affected, ☐ Moderately affected, ☐ Not affected ☐

8. Have you been diagnosed with any other chronic diseases or disorders? No ☐ Yes ☐

If yes please give more details.

☐

9. Do you have any osteochondromas/exostoses/bony bumps in your jaws/mouth?

Upper jaw No ☐ Yes ☐

If yes please give more details

☐

Lower jaw No ☐ Yes ☐
If yes please give more details.

10. How often do you see a dentist?

11. Are your dental visits covered by insurance? No ☐ Yes ☐

12. How many natural teeth do you have at present?
You can refer to picture above.
Upper jaw # Lower jaw #

13. How many sound and untreated natural teeth do you have? (meaning no fillings or dental work done or needed to be done now on these teeth.)
Upper jaw # Lower jaw #

14. Have you had any teeth removed?
Upper jaw # Lower jaw #
If you had teeth removed, how many for what reason?

15. Do you have malformed or displaced teeth? No ☐ Yes ☐
a.) Abnormal shape? (e.g. extra/missing, buckles, fused) No ☐ Yes ☐
Details

b.) Out of line? No ☐ Yes ☐
Details

c.) Other (please indicate)

16. Did your dentist ever refer to "abnormal enamel"? No ☐ Yes ☐

17. How often do you experience toothache?
Never ☐ Sometimes ☐ Often ☐

18. Do you have bleeding gingival (gums)? No ☐ Yes ☐

19. Have you been told by your dentist you have Gingivitis? No ☐ Yes ☐

20. Any other dental issues?

21. Any other comments:

If possible please send copies of photos and dental x-rays to Sarah Ziegler Email SarahZiegler@MHEResearchFoundation.org or Postal mail

Attention
Sarah Ziegler
149-34 16th Road
Whitestone, NY 11357

Respondents name:

Name of parent or guardian if this questionnaire is being submitted for a minor:

Email address:

Your home mailing address:

Your phone number and or cell phone number:

I understand all personal identifiers/contact information will be removed and coded in order to keep my anonymity / confidentiality before responses are sent to the Leiden University Medical Center for analyses.

Yes ☐

I understand my personal identifiers/contact information will remain solely with the MHE Research Foundations National MHE Research Registry. I understand my personal identifiers/contact information will not for any reason be given/transferred without my written consent.

Yes ☐

May Sarah Ziegler contact you on behalf of the Leiden University Medical Center with additional questions?

No ☐ Yes ☐
